# Supplementary material for: Comparing five generative AI chatbots’ answers to LLM-generated clinical questions with medical information scientists’ evidence summaries
Source: J Med Libr Assoc. 2026 Apr 13;114(2):94–104. doi: 10.5195/jmla.2026.2333 (PMC13075580; doi:10.5195/jmla.2026.2333)
Supplement: Supplementary file 1 — Appendix A: CHART Checklist and Methodological Diagram [file jmla-114-2-94-s01.docx]

**Appendix A. CHART Checklist and Methodological Diagram**

| HEADING | # | CHART CHECKLIST ITEM | Page #* |
| --- | --- | --- | --- |
| Title & Abstract |  |  |  |
| Title | **1a** | State that the study is assessing one or more generative AI-driven chatbots for clinical evidence or health advice. | 1 |
| Abstract/Summary | **1b** | Apply a structured format, if applicable. | 1 |
| Introduction |  |  |  |
| Background | **2a** | State the scientific background, rationale, and healthcare context for evaluating the generative AI-driven chatbot(s), referencing relevant literature when applicable. | 1-3 |
|  | **2b** | State the aims and research questions including the target audience, intervention, comparator(s), and outcome(s). | 3 |
| Methods |  |  |  |
| Model Identifiers | **3a** | State the name and version identifier(s) of the generative AI model(s) and chatbot(s) under evaluation, as well as their date of release or last update. | 4 |
|  | **3b** | State whether the generative AI model(s) and chatbot(s) are open-source or closed-source/proprietary. | 4 |
| Model Details | **4a** | State whether the generative AI model was a base model or a novel base model, tuned model, or fine-tuned model. | 4 |
|  | **4b** | If a base model is used, cite its development in sufficient detail to identify the model. | 4 |
|  | **4c** | If a novel base model, tuned model, or fine-tuned model is used, describe the pre- and/or post-implementation/deployment data and parameters. | N/A |
| Prompt Engineering | **5a** | Describe the evolution of study prompt development. | 4-6, Appendixes C-E |
|  | **5ai** | Describe the sources of prompts. | 4 |
|  | **5aii** | State the number and characteristics of the individual(s) involved in prompt engineering. | 4 |
|  | **5aiii** | Provide details of any patient and public involvement during prompt engineering. | N/A |
|  | **5b** | Provide study prompts. | Appendixes  C-E |
| Query Strategy | **6a** | State route of access to generative AI model. | 3-4 |
|  | **6b** | State the date(s) and location(s) of queries for the generative AI-driven chatbot(s) including the day, month, and year as well as city and country. | Appendix A |
|  | **6c** | Describe whether prompts were input into separate chat session(s). | 4 |
|  | **6d** | Provide all generative AI-driven chatbot output/responses | 16 (via Data Availability Statement) |
| Performance Evaluation | **7a** | Define the ground truth or reference standard used to define successful generative AI-driven chatbot performance. | N/A – the study did not use a “reference standard.” This is described on pgs. 3, 6, and 12 |
|  | **7b** | Describe the process undertaken for generative AI-driven chatbot performance evaluation. | 6-7 |
|  | **7bi** | State the number and characteristics of team members involved in performance evaluation. | 2, 6-7 |
|  | **7bii** | Provide details of any patients and public involvement during the evaluation process. | N/A |
|  | **7biii** | State whether evaluators were blinded to the identity of the generative AI-driven chatbot(s) under assessment. | 6, 14 |
| Sample Size | **8** | Report how the sample size was determined. | 7-8 |
| Data Analysis | **9a** | Describe statistical analysis methods, including any evaluation of reproducibility of generative AI-driven chatbot responses. | 8, 14 |
|  | **9ai** | Report the measures used for performance evaluation. | 6-8 |
| Results |  |  |  |
|  | **10a** | Report the alignment between generative AI-driven chatbot output and ground truth or reference standard using quantitative or mixed methods approaches as applicable. | Alignment is reported on pgs. 9-11. Note: there was not a true “reference standard” in the study. |
|  | **10b** | For responses deviating from the ground truth or reference standard, state the nature of the difference(s). | N/A - There was not a true “reference standard” in the study. |
|  | **10c** | Report the assessment for potentially harmful, biased, or misleading responses. | 13 |
| Discussion |  |  |  |
|  | **11a** | Interpret study findings in the context of relevant evidence. | 11-15 |
|  | **11b** | Describe the strengths and limitations of the study. | 11-15 |
|  | **11c** | Describe the potential implications for practice, education, policy, regulation, and research. | 11-15 |
| Open Science |  |  |  |
| Disclosures | **12a** | Report any relevant conflicts of interest for all authors. | 16 |
| Funding | **12b** | Report sources of funding and their role in the conduct and reporting of the study. | 16 |
| Ethics | **12c** | Describe the process undertaken for ethical approval. | 3 |
|  | **12ci** | Describe the measures taken to safeguard data privacy of patient health information, as applicable. | N/A |
|  | **12cii** | State whether permission/licensing was obtained for the use of original, copyrighted data. | N/A |
| Protocol | **12d** | Provide a study protocol. | N/A |
| Data availability | **12e** | State where study data, code repository, and model parameters can be accessed. | 16 |

*If in supplementary appendix, indicate “supp” and appendix #, if applicable.

*From:* The CHART Collaborative; Huo B, Collins GS, Chartash D, Thirunavukarasu AJ, Flanagin A, et al. Reporting guideline for chatbot health advice studies: the CHART statement. JAMA Netw Open. 2025 Aug 1;8(8):e2530220. DOI: <https://doi.org/10.1001/jamanetworkopen.2025.30220>

CHART Methodological Diagram

**Total generative AI-driven chatbot(s) (n=5)**

**Name(s) & version identifier(s): ChatGPT-4o, Gemini 2.0 Flash, DeepSeek R1, Grok-3, Copilot (version not specified)**

Open-source (n=1)

Closed-source/proprietary (n=4)

Subscription (n=0)

API (n=0)

Other (n=4 web-based free versions)

Base model (n=5)

Novel base model (n=0)

Tuned model (n=0)

Fine-tuned model (n=0)

**Prompt Engineering**

Prompt engineers (n=7)

Investigator-derived (n=3)

Clinician-derived (n=0)

Patient-derived (n=0)

Prompt sources (n=1)

Total prompts (n=3)

Follow-up prompts (n=2)

**Query**

Queries to obtain key elements:

Date(s) of query (06/12/2025-06/18/2025)

Chat sessions (n=450)

Location(s): Franklin, TN, USA

Language(s): English

Queries to obtain PICO questions:

Date(s) of query (11/11/2024-11/12/2024)

Chat sessions (n=53)

Location(s): Franklin, TN, USA

Language(s): English

Queries to obtain LLM answers:

Date(s) of query (01/24/2025-04/24/2025)

Chat sessions (n=225)

Location(s): Tampa, FL, USA

Language(s): English

**Performance Evaluation**

Repeat queries (n=0)

Date(s) of query (N/A)

Location(s): N/A

Discrepancies: N/A

Evaluators (n=4)

Patient/public (n=0)

Automated (n=0)

Model output/responses (n=450)

Valid (n=450)

Missing/invalid (n=0)

**Reproducibility**
