# Supplementary material for: Comparing five generative AI chatbots’ answers to LLM-generated clinical questions with medical information scientists’ evidence summaries
Source: J Med Libr Assoc. 2026 Apr 13;114(2):94–104. doi: 10.5195/jmla.2026.2333 (PMC13075580; doi:10.5195/jmla.2026.2333)
Supplement: Supplementary file 4 — Appendix D: Prompt for Submitting the Questions to the LLMs [file jmla-114-2-94-s04.docx]

**Appendix D. Prompt for Submitting the Questions to the LLMs**

**#CONTEXT#**

I am a medical librarian at a major academic health sciences center. In my team, our members provide evidence-based filtered summaries of the biomedical literature for use in patient care.

**#OBJECTIVE#**

Your task is to provide a summary of evidence that answers a clinical question I will provide to you. This involves scanning both the published and grey literature. The aim is to create a narrative statement that answers the question. When possible, the narrative statement should comment on the strengths and weaknesses of the evidence. Only use information that was available prior to [date of packet].

**#STYLE#**

Write in an objective, professional, and educational style in the role of a medical librarian. Write the response in a style that is directed towards medical professionals interested in understanding the available evidence.

**#TONE#**

Maintain a balanced and objective tone throughout the summary.

**#AUDIENCE#**

The target audience is clinicians providing patient care. Assume a readership that has direct experience in taking care of patients.

**#RESPONSE FORMAT#**

Provide an easy-to-follow narrative summary in paragraph format.

**#START ANALYSIS#**

If you understand, ask me to enter the clinical question.

*Note:* This prompt was reused from our team’s previous study [1] and developed based on the COSTAR framework [2].

**References:**

1. Blasingame MN, Koonce TY, Williams AM, Giuse DA, Su J, Krump PA, Giuse NB. Evaluating a large language model's ability to answer clinicians' requests for evidence summaries. J Med Libr Assoc. 2025 Jan 14;113(1):65-77. DOI: <https://doi.org/10.5195/jmla.2025.1985>.
2. GovTech Data Science & AI Division. Prompt engineering playbook (Beta v3) [Internet]. Singapore Government Developer Portal. 2023 [cited 24 Sept 2025]. <<https://www.developer.tech.gov.sg/products/collections/data-science-and-artificial-intelligence/playbooks/prompt-engineering-playbook-beta-v3.pdf>>.
