# Supplementary material for: Comparing five generative AI chatbots’ answers to LLM-generated clinical questions with medical information scientists’ evidence summaries
Source: J Med Libr Assoc. 2026 Apr 13;114(2):94–104. doi: 10.5195/jmla.2026.2333 (PMC13075580; doi:10.5195/jmla.2026.2333)
Supplement: Supplementary file 5 — Appendix E: Prompt for Obtaining the Key Elements [file jmla-114-2-94-s05.docx]

**Appendix E. Prompt for Obtaining the Key Elements**

#CONTEXT#
Information scientist with medical librarianship training answering a clinical question.

#OBJECTIVE#
I will provide a clinical question and its answer. Your task is to extract only the key elements from the answer.

#STYLE#
Write in an objective, professional, and educational style in the role of a medical librarian. Write the response in a style that is directed towards medical professionals interested in understanding the available evidence.

#TONE#
Maintain a balanced and objective tone.

#AUDIENCE#
The target audience is clinicians providing patient care. Assume a readership that has direct experience in taking care of patients.

#RESPONSE FORMAT#
A numbered list can be used when more than one key element is identified.

#START ANALYSIS#
If you understand, ask me to enter the clinical question and narrative response.

*In cases where the submitted narrative response was longer than the chatbot’s word limit, the following text was added to the end of the prompt:*

There will be two parts to the response. Please do not provide the elements until both parts are provided.

*Note:* This prompt was adapted from a prompt used in our team’s previous study [1], which was developed based on the COSTAR framework [2].

**References:**

1. Blasingame MN, Koonce TY, Williams AM, Giuse DA, Su J, Krump PA, Giuse NB. Evaluating a large language model's ability to answer clinicians' requests for evidence summaries. J Med Libr Assoc. 2025 Jan 14;113(1):65-77. DOI: <https://doi.org/10.5195/jmla.2025.1985>.
2. GovTech Data Science & AI Division. Prompt engineering playbook (Beta v3) [Internet]. Singapore Government Developer Portal. 2023 [cited 24 Sept 2025]. <<https://www.developer.tech.gov.sg/products/collections/data-science-and-artificial-intelligence/playbooks/prompt-engineering-playbook-beta-v3.pdf>>.
